# Symptom improvement with mirabegron treatment is associated with urobiome changes in adult women

**DOI:** 10.1007/s00192-022-05190-w

**Published:** 2022-04-12

**Authors:** Thomas Halverson, Elizabeth R. Mueller, Linda Brubaker, Alan J. Wolfe

**Affiliations:** 1 Department of Microbiology and Immunology, Stritch School of Medicine, Loyola University Chicago, Maywood, IL 60153 USA; 2 Division of Female Pelvic Medicine and Reconstructive Surgery, Departments of Urology & Obstetrics/Gynecology, Loyola University Medical Center, Maywood, IL USA; 3 Department of Obstetrics, Gynecology and Reproductive Sciences, University of California San Diego, La Jolla, CA USA

**Keywords:** Expanded quantitative urine culture, Microbiome, Mirabegron, Overactive bladder, Urgency urinary incontinence, Urobiome

## Abstract

**Introduction and hypothesis:**

Mirabegron, a beta-3 agonist, is prescribed for urgency urinary incontinence (UUI). We assessed the correlation of symptom improvement with urobiome characteristics in adult women participants prescribed mirabegron for UUI treatment.

**Methods:**

We enrolled participants seeking UUI treatment who selected mirabegron and agreed to participate in this 12-week, open label study conducted at the Female Pelvic Medicine and Reconstructive Surgery Center at Loyola University Medical Center. Following eligibility screening and research consent, participants completed the overactive bladder questionnaire (OAB-Q) and provided a catheterized urine sample at baseline, 4, 8, and 12 weeks. The primary outcome, symptom improvement at 12 weeks, was based on the validated Patient Global Symptom Control questionnaire score to dichotomize symptom response (responder vs nonresponder [PGSC score ≤3]). Urine samples were processed by the Expanded Quantitative Urine Culture (EQUC) protocol.

**Results:**

Eighty-three participants (mean age 68 years) completed baseline assessment. Of the 47 participants with primary outcome data and samples analysis, there were 16 responders and 31 nonresponders; responder groups were similar demographically. Living microbes were detected in most participants. There were no significant differences in alpha diversity (within sample) at baseline between groups. However, at the 12-week follow-up, the responder urobiome became significantly richer, with a larger number of genera (*p* = 0.027) and was significantly more diverse than the nonresponders.

**Conclusions:**

Longitudinal urobiome changes are associated with symptom improvement in adult women being treated with mirabegron for UUI. The mechanism for symptoms improvement may relate to the detected changes in the urobiome and warrants further study.

## Introduction

Understanding of overactive bladder (OAB) and urinary urgency incontinence (UUI) pathophysiology is evolving and includes a broader consideration of sensory-motor etiologies related to bladder control. This evolution of knowledge is augmented by the growing awareness of the urinary microbiome (urobiome). Given data correlating the urobiome with treatment response, researchers have explored the possibility of microbes or microbial products as therapeutic targets for OAB/UUI symptoms or as etiological factors. In 2016, Thomas-White et al. provided evidence that UUI symptom response after 12 weeks of solifenacin was associated with pre-treatment differences in the urobiome [1]. Compared with those who never responded to their medication and those who required an increased dose, low-dose responders had a significantly less diverse urobiome prior to treatment.

Mirabegron is an FDA-approved beta-3 agonist for treatment of UUI. As prior research has focused on the urobiome response to traditional anticholinergic therapy [1], little is known about predictors of symptom control with mirabegron. In this study, we assessed baseline urobiome characteristics associated with treatment response and changes over 12 weeks in participants starting clinically indicated mirabegron for UUI treatment.

## Materials and methods

### Study design and population

Following Institutional Review Board approval (LU207102), we enrolled participants in this 12-week study between December 2015 and December 2018 from the Female Pelvic Medicine and Reconstructive Surgery Center at Loyola University Medical Center. Potential participants were recruited from adult women seeking UUI treatment. An individual was potentially eligible for this 12-week, open label study following eligibility screening using the long version of the validated symptom questionnaire, the Pelvic Floor Distress Inventory (PFDI) [2, 3] once her physician had made a clinical decision to prescribe mirabegron and she accepted a clinical prescription for mirabegron with the intent to begin the treatment.

Exclusion criteria included current or suspected urinary tract infection (UTI; based on urine dipstick), history of recurrent UTI, antibiotic exposure in the past 4 weeks for any reason, immunological deficiency, neurological disease known to affect the lower urinary tract, pelvic malignancy or radiation, untreated symptomatic pelvic organ prolapse (POP) greater than POP-Q stage II (vaginal protrusion more than 1 cm outside of the vaginal hymen) or pregnancy.

Participants gave verbal and written research consent for chart abstraction and urine collection with analysis for research purposes. Enrolled participants also completed the overactive bladder questionnaire (OAB-Q) [4]. As part of normal clinical care, participants provided a catheterized urine sample prior to UUI treatment with mirabegron, which was provided at no cost for 12 weeks. The drug manufacturer (Astellas) provided the medication but had no role in the study design, conduct, analysis, or interpretation. Participants with UUI also agreed to provide three additional catheterized urine samples at 4, 8, and 12 weeks during UUI treatment for analysis.

The primary outcome was symptomatic response at 12 weeks, based on the validated Patient Global Symptom Control (PGSC) questionnaire score, which consists of a single question that addresses improvement following treatment [5]. Participants with PGSC scores of 4 or 5 were defined as “responders”; those with PGSC scores ≤3 were considered “nonresponders.” The starting mirabegron dose was 25 mg. At 4 weeks, nonresponders were offered a dosage increase to 50 mg at 4 weeks. Participants who were intolerant of the medication could withdraw at any point during the study. The research team assessed adverse events at normally scheduled visits, and at other intervals during participant-initiated contact.

### Sample collection and microbe detection

Urine was collected aseptically via transurethral catheter and a portion placed in a BD Vacutainer Plus C&S preservative tube for culture. Samples were processed according to the Expanded Quantitative Urine Culture (EQUC) protocol [6]. EQUC uses 0.1 ml of urine spread quantitatively onto BAP, Chocolate and Colistin, Nalidixic Acid (CNA) agars (BD BBL™ Prepared Plated Media), then incubated in 5% CO_2_ (37°C for 48 h). A second set of BAPs were inoculated with 0.1 ml of urine and incubated in room atmosphere at 37°C for 48 h. In addition, 0.1 ml of urine was inoculated onto CDC Anaerobe 5% sheep blood agar (ABAP) plates (BD BBL™ Prepared Plated Media) and incubated under anaerobic conditions at 37°C for 48 h. The detection level was 10 CFU/ml, represented by 1 colony of growth on any of the plates. Each morphologically distinct colony type was isolated on a different plate of the same media to prepare a pure culture that was used for microbe identification. Matrix-assisted laser desorption ionization time of flight mass spectrometry with the MALDI Biotyper 3.0 software (Bruker Daltonics, Billerica, MA, USA) was used to identify the bacterial isolates. To assess the overall diversity of the population by total number of unique species isolated, a species accumulation curve was generated.

### Data analyses

Standard statistical methods were used to compare participant demographics and symptom data between participants who did or did not provide primary outcome data, as well as 12-week responders versus nonresponders. Continuous variables are reported as means and standard deviations (SD) or medians and interquartile ranges (IQR) and categorical variables are reported as frequencies and percentages. Participants without 12-week data were excluded from the longitudinal analyses. Fisher’s exact and Kruskal–Wallis tests were used to compare demographic and culture (e.g., abundance and diversity) information among UUI response groups. For cultured microbes, abundance was measured using total CFU/ml. Within sample (alpha) diversity was calculated in RStudio v1.2.1335 (RStudio, Boston, MA, USA). Genera richness or numbers of taxa per sample, evenness, or distribution of taxa within a sample (Pielou), and combinations of richness and evenness (Inverse Simpson and Shannon). Post hoc pairwise comparisons were made using Wilcoxon Rank sum tests for continuous variables with significant overall *p* values. All statistical analyses were conducted using SPSS software version 19 or SAS software v9.4 (SAS Institute, Cary, NC, USA). Significance was assessed at an alpha level of 0.05. Results were not adjusted for multiple comparisons as the analyses are considered descriptive.

## Results

### Population description over the course of the study

**Fig. 1:**
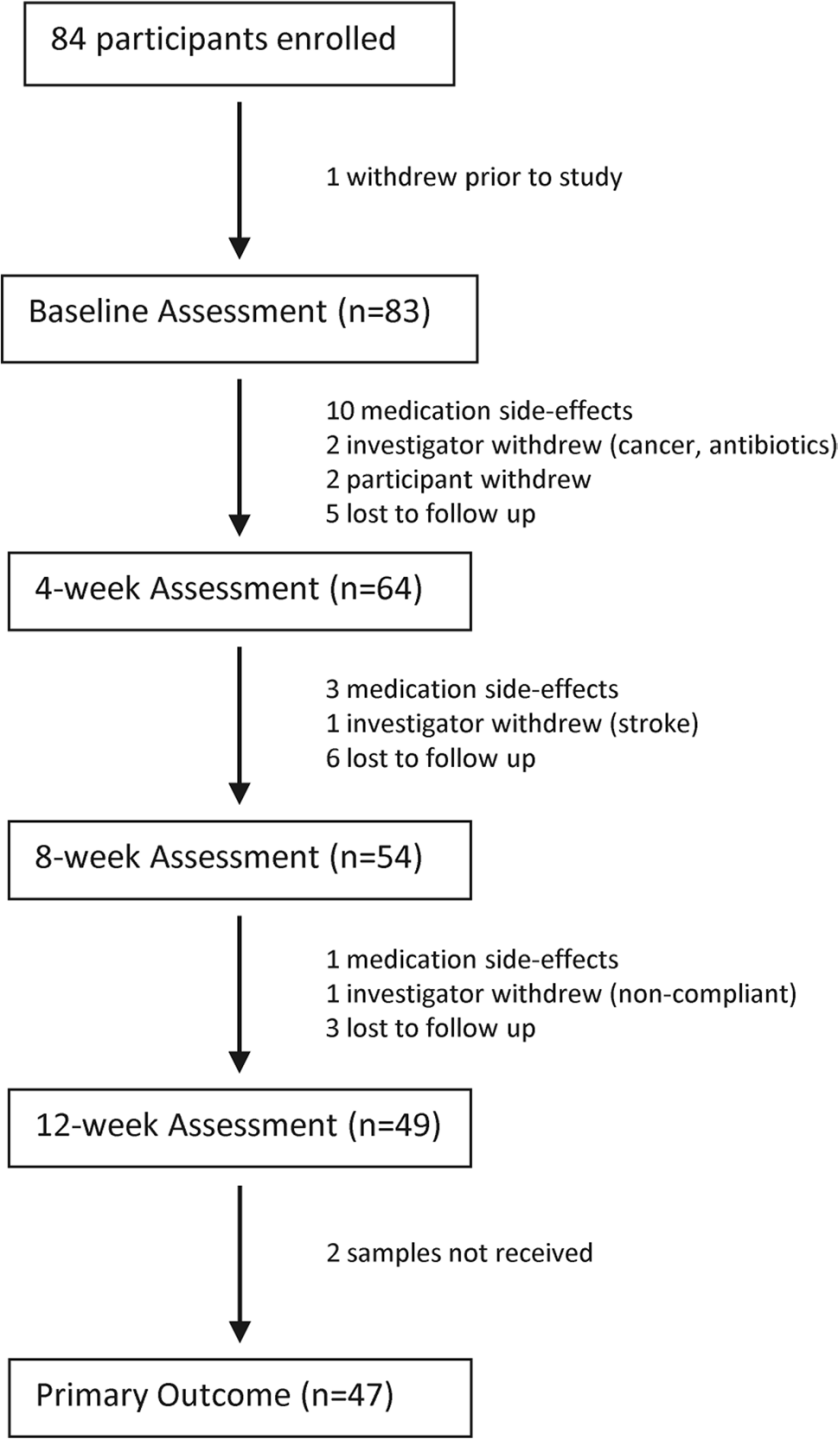
Consolidated Standards of Reporting Trials flow diagram

Table 1 displays the baseline demographics and characteristics of the study population. Eighty-three of the 84 enrolled participants provided complete baseline assessment (one withdrew prior to baseline assessment). These participants had a mean age of (68 years); most were white (80%). Figure 1 displays the flow of participants through the 12-week study period. Primary outcome (symptom improvement at 12 weeks) as well as laboratory data were available for 47 participants (57%). There were no significant baseline demographic differences between those who completed the study and those who did not provide primary outcome data (Table 1). The baseline symptom severity was similar in completers and in those who were withdrawn or voluntarily withdrew prior to 12 weeks. However, we note a trend of over-representation of nonsmokers in the noncompleter group (*p* = 0.07).

**Table 1:** Baseline demographics and clinical characteristics by study population and response category

	Total (*N* = 83)	12-week outcome (*N* = 47)	No primary outcome (*N* = 36)	*p* value*	Total 12-week outcomes (*N* = 47)	Responders (*N*N = 16)	Nonresponders (*N* = 31)	*p* value*
Demographics								
Age (years)	68.0 (57.0–76.0)	68.0 (55.0–73.0)	69.0 (55.0–77.0)	0.11	67.0 (56.0–75.0)	64.5 (56.0–72.5)	69.0 (55.0–77.0)	0.39
Body mass index (kg/m^2^)	31.7 (25.8–39.6)	31.7 (25.7–39.6)	32.1 (26.6–39.9)	0.73	31.7 (25.8–38.5)	28.4 (25.6–37.0)	32.1 (26.4–39.9)	0.50
Race				0.70				0.37
Black	12 (14%)	6 (13%)	6 (17%)		6 (13%)	1 (6%)	5 (16%)	
White	66 (80%)	39 (83%)	27 (75%)		38 (81%)	13 (81%)	25 (81%)	
Other	5 (6%)	2 (4%)	3 (8%)		3 (6%)	2 (13%)	1(3%)	
Estrogen status				0.38				0.35
Estrogen user	5 (6%)	2 (4%)	3 (8%)		2 (4%)	0 (0%)	2 (6%)	
Not an estrogen user	71 (86%)	42 (89%)	28 (78%)		42 (89%)	14 (88%)	28 (90%)	
Not reported	7 (8%)	3 (6%)	5 (14%)		3 (6%)	2 (13%)	1 (3%)	
Hormone therapy	7 (8%)	5 (11%)	2 (6%)	0.69	5 (11%)	1 (6%)	4 (13%)	0.65
Diabetes	16 (19%)	8 (17%)	8 (22%)	0.55	8 (17%)	3 (19%)	5 (16%)	1.0
Smoker	8 (10%)	2 (4%)	6 (17%)	0.07	3 (6%)	1 (6%)	2 (6%)	1.0
High blood pressure	43 (52%)	25 (53%)	18 (50%)	0.77	24 (51%)	5 (31%)	19 (61%)	0.066
Heart disease	12 (14%)	7 (15%)	5 (14%)	0.90	7 (15%)	1 (6%)	6 (19%)	0.40
COPD	4 (5%)	1 (2%)	3 (8%)	0.31	1 (2%)	0 (0%)	1 (3%)	1.0
Asthma	13 (17%)	6 (13%)	7 (19%)	0.43	6 (13%)	1 (6%)	5 (16%)	0.65
Symptoms								
Symptom score^a^	66.3 (50.0–85.0)	67.5 (55.0–87.5)	65.0 (45.0–77.5)	0.12	70 (±18)	59 (±18)	76 (±15)	*0.002*
Coping score^a^	51.3 (17.5–75.0)	52.5 (15.0–77.5)	42.5 (17.5–72.5)	0.99	52.5 (15.0–77.5)	76.3 (56.3–87.5)	40.0 (12.5–62.5)	*0.007*
Health-related quality of life^a^	54.4 (23.2–74.4)	54.4 (28.8–74.4)	50.4 (23.2–74.7)	0.97	54.4 (23.2–74.4)	74.8 (65.6–79.2)	46.4 (18.4–64.0)	*0.001*
Urinary Distress Inventory^b^	112.0 (81.0–153.0)	115.0 (81.7–162.0)	10.9 (68.1–150.0)	0.70	115.0 (81.7–162)	75.5 (45.9–119.0)	117.0 (89.7–187.0)	*0.011*
Pelvic Organ Prolapse Distress Inventory^b^	58.9 (33.3–105.0)	52.7 (29.8–102.0)	77.4 (40.5–107.0)	0.26	55.4 (31.1–101)	41.7 (26.8–71.7)	61.9 (39.9–123.0)	0.086
Colorectal-Anal Distress Inventory^b^	71.2 (28.6–134.0)	71.4 (30.7–127.0)	69.4 (28.2–136.0)	0.95	71.5 (30.7–127)	53.6 (27.4–99.2)	77.0 (30.7–160.0)	0.16

*COPD* chronic obstructive pulmonary disease

*Pearson’s Chi-squared and Fisher’s exact test were used with categorical variables. Student’s *t* test was used with continuous variables. Italics indicate significant at the 0.05 threshold

^a^Based on the Overactive Bladder questionnaire

^b^Based on the Pelvic Floor Disease Inventory

Sixteen of the 47 participants who completed the study were responders; the remaining 31 were nonresponders. One participant had sufficient symptom control with the 25-mg dose; all others utilized the 50-mg dosing regimen. Age, BMI, race, estrogen use status, and other clinical factors did not differ between responders and nonresponders. Responders had significantly better Symptom, Coping, Health-Related Quality of Life, and Urinary Distress Inventory scores (Table 1, *p* < 0.05, Kruskal–Wallis test).

### Urobiome characteristics

Living microbes were detected by EQUC in most participants: 75 (90%) at baseline and 41 (87%) at 12 weeks. At baseline, there was no significant difference between the responders and the nonresponders with respect to alpha diversity measures. However, at the 12-week follow-up, the urobiome of responders was significantly more diverse than the nonresponders, as measured by the Shannon diversity index (*p* = 0.035; Table 2). This increased diversity in responders appears to be related to a trend toward increased richness compared with nonresponders (*p* = 0.075; Table 2). Between baseline and the 12-week follow-up, the urobiome of responders became significantly richer, with a larger number of genera (*p* = 0.027; Table 3).

**Table 2:** Diversity measures over time by response category in those who completed the study (*n* = 47)

		Baseline		12-week	
Measure	Group	Median (IQR)	*p* value	Median (IQR)	*p* value
Shannon	Responder	0.47 (0.0077–0.91)	0.88	0.77 (0.42–1.06)	*0.035*
	Nonresponder	0.53 (0–1.10)		0.17 (0.012–1.07)	
Simpson	Responder	0.38 (0.0085–0.58)	0.31	0.43 (0.22–0.59)	0.37
	Nonresponder	0.50 (0.24–0.72)		0.22 (0.011–0.69)	
Genera richness	Responder	3 (2–4)	0.98	5 (3–7)	0.075
	Nonresponder	3 (1–5)		4 (2–6)	
Pielou's evenness	Responder	0.52 (0.028–0.68)	0.24	0.58 (0.31–0.81)	0.24
	Nonresponder	0.75 (0.33–1)		0.32 (0.035–0.80)	

Italics indicate significant at the 0.05 threshold

**Table 3:** Diversity measures of response category by timepoint in those who completed the study (*n* = 47)

		Responder		Nonresponder	
Measure	Timepoint	Median (IQR)	*p* value	Median	*p* value
Shannon	Baseline	0.47 (0.0077–0.91)	0.12	0.53 (0–1.10)	0.77
	12-week	0.77 (0.42–1.06)		0.17 (0.012–1.07)	
Simpson	Baseline	0.38 (0.0085–0.58)	0.58	0.50 (0.24–0.72)	0.23
	12-week	0.43 (0.22–0.59)		0.22 (0.011–0.69)	
Genera richness	Baseline	3 (2–4)	*0.0.27*	3 (1–5)	0.48
	12-week	5 (3–7)		4 (2–6)	
Pielou's evenness	Baseline	0.52 (0.028–0.68)	0.58	0.75 (0.33–1)	0.12
	12-week	0.58 (0.31–0.81)		0.32 (0.035–0.80)	

Italics indicate significant at the 0.05 threshold

For the 47 completers, regardless of response group, bacterial detection frequency at the genus level was similar at baseline and at 12 weeks, although the genus *Aerococcus* may be more common in the responders at 12 weeks (*p* = 0.068; Table 4). At baseline, the two response groups had similar bacterial detection frequency at the species level. However, at 12 weeks, differences were detected. Responders were more likely to have the species *Actinomyces neuii* (*p* = 0.036), *Corynebacterium aurimucosum* (*p* = 0.013), *Lactobacillus gasseri* (*p* = 0.027), and perhaps *Streptococcus anginosus* (*p* = 0.057; Table 5).

**Table 4:** Frequency of detection (genera) from a catheterized urine sample at baseline and at 12 weeks by responder category

Genus	Baseline			12-week		
	Responder (*n* = 15)	Nonresponder (*n* = 31)	*p* value	Responder (*n* = 16)	Nonresponder (*n* = 31)	*p* value
*Actinobaculum*				1 (6.25%)	2 (6.45%)	1
*Actinomyces*	3 (20%)	8 (25.81%)	1	6 (37.5%)	5 (16.13%)	0.15
*Actinotignum*	0 (0%)	1 (3.23%)	1	2 (12.5%)	2 (6.45%)	0.60
*Aerococcus*	3 (20%)	7 (22.58%)	1	6 (37.5%)	4 (12.9%)	0.068
*Alloscardovia*	2 (13.33%)	2 (6.45%)	0.59	2 (12.5%)	2 (6.45%)	0.50
*Arthrobacter*	1 (6.67%)	3 (9.68%)	1	2 (12.5%)	3 (9.68%)	1
*Bifidobacterium*	2 (13.33%)	2 (6.45%)	0.59	3 (18.75%)	1 (3.23%)	0.11
*Brevibacterium*	0 (0%)	2 (6.45%)	1	0 (0%)	2 (6.45%)	0.54
*Candida*	1 (6.67%)	0 (0%)	0.33	0 (0%)	3 (9.68%)	0.54
*Corynebacterium*	5 (33.33%)	11 (35.48%)	1	8 (50.0%)	10 (32.26%)	0.34
*Dermabacter*				0 (0%)	1 (3.23%)	1
*Dialister*	0 (0%)	1 (3.23%)	1			
*Enterobacter*				0 (0%)	1 (3.23%)	1
*Enterococcus*	0 (0%)	1 (3.23%)	1	4 (25.0%)	8 (25.81%)	1
*Escherichia*	3 (20%)	7 (22.58%)	1	4 (25.0%)	8 (25.81%)	1
*Facklamia*				3 (18.75%)	2 (6.45%)	0.32
*Gardnerella*	1 (6.67%)	4 (12.9%)	1	3 (18.75%)	9 (29.03%)	0.73
*Globicatella*	1 (6.67%)	1 (3.23%)	1			
*Haemophilus*	1 (6.67%)	0 (0%)	0.33			
*Klebsiella*	1 (6.67%)	1 (3.23%)	1	0 (0%)	1 (3.23%)	1
*Kocuria*				1 (6.25%)	0 (0%)	0.34
*Lactobacillus*	6 (40%)	13 (41.94%)	1	10 (62.5%)	14 (45.16%)	0.36
*Neisseria*	0 (0%)	1 (3.23%)	1			
*Oligella*				0 (0%)	1 (3.23%)	1
*Propionibacterium*				1 (6.25%)	1 (3.23%)	1
*Proteus*	1 (6.67%)	0 (0%)	0.33			
*Pseudomonas*	0 (0%)	1 (3.23%)	1			
*Staphylococcus*	4 (26.67%)	9 (29.03%)	1	7 (43.75%)	11 (35.48%)	0.75
*Stenotrophomonas*				0 (0%)	1 (3.23%)	1
*Streptococcus*	4 (26.67%)	14 (45.16%)	0.34	11 (68.75%)	14 (45.16%)	0.22
*Veillonella*				0 (0%)	2 (6.45%)	0.54
*Weeksella*	1 (6.67%)	0 (0%)	0.33			
Undetectable	1 (6.67%)	5 (16.13%)	0.65	0 (0%)	3 (9.68%)	0.54

Blank indicates not detected

Bonferroni adjusted *p* value 0.002

**Table 5:** Frequency of detection (species) from catheterized urine samples at baseline and 12 weeks by responder category^a^

Species	Baseline			12-week		
	Responder (*n* = 15)	Nonresponder (*n* = 31)	*p* value	Responder (*n* = 16)	Nonresponder (*n* = 31)	*p* value
*Actinobaculum massiliense*				1 (6.25%)	2 (6.45%)	1
*Actinomyces europaeus*				0 (0%)	2 (6.45%)	0.54
*Actinomyces neuii*	1 (6.67%)	5 (16.13%)	0.65	5 (31.25%)	2 (6.45%)	*0.036*
*Actinomyces odontolyticus*	0 (0%)	1 (3.23%)	1			
*Actinomyces radingae*	0 (0%)	2 (6.45%)	1			
*Actinomyces turicensis*	1 (6.67%)	2 (6.45%)	1	1 (6.25%)	1 (3.23%)	1
*Actinomyces urogenitalis*	1 (6.67%)	0 (0%)	0.33			
*Actinotignum sanguinis*				0 (0%)	1 (3.23%)	1
*Actinotignum schaalii*	0 (0%)	1 (3.23%)	1	2 (12.5%)	1 (3.23%)	0.25
*Aerococcus christensenii*				1 (6.25%)	0 (0%)	0.34
*Aerococcus sanguinicola*	1 (6.67%)	2 (6.45%)	1	1 (6.25%)	1 (3.23%)	1
*Aerococcus urinae*	3 (20%)	6 (19.35%)	1	5 (31.25%)	3 (9.68%)	0.10
*Aerococcus viridans*	0 (0%)	1 (3.23%)	1			
*Alloscardovia omnicolens*	2 (13.33%)	2 (6.45%)	0.59	2 (12.5%)	2 (6.45%)	0.60
*Arthrobacter cumminsii*	1 (6.67%)	3 (9.68%)	1	2 (12.5%)	3 (9.68%)	1
*Bifidobacterium breve*	2 (13.33%)	2 (6.45%)	0.59	2 (12.5%)	1 (3.23%)	0.26
*Bifidobacterium scardovii*				1 (6.25%)	0 (0%)	0.34
*Brevibacterium paucivorans*	0 (0%)	1 (3.23%)	1			
*Brevibacterium ravenspurgense*	0 (0%)	2 (6.45%)	1	0 (0%)	2 (6.45%)	0.54
*Candida albicans*				0 (0%)	3 (9.68%)	0.54
*Candida glabrata*	1 (6.67%)	0 (0%)	0.33	0 (0%)	1 (3.23%)	1
*Corynebacterium afermentans*				1 (6.25%)	0 (0%)	0.34
*Corynebacterium amycolatum*	2 (13.33%)	3 (9.68%)	1	3 (18.75%)	5 (16.13%)	1
*Corynebacterium aurimucosum*	2 (13.33%)	2 (6.45%)	0.59	6 (37.5%)	2 (6.45%)	0.011
*Corynebacterium coyleae*	2 (13.33%)	4 (12.9%)	1	3 (18.75%)	2 (6.45%)	0.32
*Corynebacterium freneyi*				0 (0%)	1 (3.23%)	1
*Corynebacterium glucuronolyticum*				1 (6.25%)	1 (3.23%)	1
*Corynebacterium imitans*	0 (0%)	1 (3.23%)	1	0 (0%)	4 (12.9%)	0.28
*Corynebacterium jeikeium*	0 (0%)	1 (3.23%)	1			
*Corynebacterium kroppenstedtii*	0 (0%)	1 (3.23%)	1			
*Corynebacterium lipophile group F1*	0 (0%)	1 (3.23%)	1	1 (6.25%)	2 (6.45%)	1
*Corynebacterium minutissimum*	0 (0%)	1 (3.23%)	1			
*Corynebacterium pyruviciproducens*				2 (12.5%)	0 (0%)	0.11
*Corynebacterium riegelii*	1 (6.67%)	2 (6.45%)	1	1 (6.25%)	2 (6.45%)	1
*Corynebacterium simulans*				1 (6.25%)	0 (0%)	0.34
*Corynebacterium sp.*				1 (6.25%)	0 (0%)	0.34
*Corynebacterium tuberculostearicum*				1 (6.25%)	0 (0%)	0.34
*Corynebacterium tuscaniense*	0 (0%)	2 (6.45%)	1	1 (6.25%)	0 (0%)	0.34
*Corynebacterium urealyticum*	0 (0%)	1 (3.23%)	1	0 (0%)	2 (6.45%)	0.54
*Dermabacter hominis*				0 (0%)	1 (3.23%)	1
*Dialister microaerophilus*	0 (0%)	1 (3.23%)	1			
*Enterobacter aerogenes*				0 (0%)	1 (3.23%)	1
*Enterococcus faecalis*	0 (0%)	1 (3.23%)	1	4 (25.0%)	8 (25.81%)	1
*Escherichia coli*	3 (20%)	7 (22.58%)	1	4 (25.0%)	8 (25.81%)	1
*Facklamia hominis*				3 (18.75%)	2 (6.25%)	0.32
*Gardnerella sp.*	0 (0%)	1 (3.23%)	1	0 (0%)	1 (3.23%)	1
*Gardnerella vaginalis*	1 (6.67%)	4 (12.9%)	1	3 (18.75%)	9 (29.03%)	0.51
*Globicatella sulfidifaciens*	1 (6.67%)	1 (3.23%)	1			
*Haemophilus parainfluenzae*	1 (6.67%)	0 (0%)	0.33			
*Klebsiella oxytoca*	0 (0%)	1 (3.23%)	1			
*Klebsiella pneumoniae*	1 (6.67%)	0 (0%)	0.33	0 (0%)	1 (3.23%)	1
*Kocuria rhizophila*				1 (6.25%)	0 (0%)	0.34
*Lactobacillus crispatus*	2 (13.33%)	2 (6.45%)	0.59	3 (18.75%)	2 (6.45%)	0.32
*Lactobacillus delbrueckii*	0 (0%)	3 (9.68%)	0.54	1 (6.25%)	2 (6.45%)	1
*Lactobacillus fermentum*	1 (6.67%)	0 (0%)	0.33			
*Lactobacillus gasseri*	3 (20%)	5 (16.13%)	1	7 (43.75%)	4 (12.9%)	*0.027*
*Lactobacillus iners*	3 (20%)	3 (9.68%)	0.38	2 (12.5%)	4 (12.9%)	1
*Lactobacillus jensenii*	3 (20%)	4 (12.9%)	0.67	4 (25.0%)	4 (12.9%)	0.42
*Lactobacillus rhamnosus*				1 (6.25%)	1 (3.23%)	1
*Lactobacillus sp.*				0 (0%)	1 (3.23%)	1
*Lactobacillus vaginalis*	0 (0%)	1 (3.23%)	1	1 (6.25%)	0 (0%)	0.34
*Neisseria subflava*	0 (0%)	1 (3.23%)	1			
*Oligella urethralis*				0 (0%)	1 (3.23%)	1
*Propionibacterium avidum*				1 (6.25%)	0 (0%)	0.34
*Propionibacterium sp.*				0 (0%)	1 (3.23%)	1
*Proteus mirabilis*	1 (6.67%)	0 (0%)	0.33			
*Pseudomonas aeruginosa*	0 (0%)	1 (3.23%)	1			
*Staphylococcus aureus*				0 (0%)	1 (3.23%)	1
*Staphylococcus capitis*	0 (0%)	1 (3.23%)	1	0 (0%)	1 (3.23%)	1
*Staphylococcus caprae*				0 (0%)	1 (3.23%)	1
*Staphylococcus epidermidis*	3 (20%)	5 (16.13%)	1	5 (31.25%)	7 (22.58%)	0.73
*Staphylococcus haemolyticus*	1 (6.67%)	3 (9.68%)	1	3 (18.75%)	1 (3.23%)	0.11
*Staphylococcus hominis*	1 (6.67%)	0 (0%)	0.33	2 (12.5%)	1 (3.23%)	0.26
*Staphylococcus lugdunensis*	0 (0%)	1 (3.23%)	1			
*Staphylococcus simulans*	0 (0%)	1 (3.23%)	1	1 (6.25%)	0 (0%)	0.34
*Stenotrophomonas maltophilia*				0 (0%)	1 (3.23%)	1
*Streptococcus agalactiae*	0 (0%)	3 (9.68%)	0.54	2 (12.5%)	1 (3.23%)	0.25
*Streptococcus anginosus*	4 (26.67%)	9 (29.03%)	1	10 (62.5%)	9 (29.03%)	0.057
*Streptococcus constellatus*				0 (0%)	1 (3.23%)	1
*Streptococcus gallolyticus*	0 (0%)	2 (6.45%)	1	0 (0%)	1 (3.23%)	1
*Streptococcus mitis*	0 (0%)	3 (9.68%)	0.54	0 (0%)	3 (9.68%)	0.54
*Streptococcus oralis*				0 (0%)	3 (9.68%)	0.54
*Streptococcus parasanguinis*	1 (6.67%)	1 (3.23%)	1	0 (0%)	1 (3.23%)	1
*Streptococcus salivarius*	0 (0%)	1 (3.23%)	1	0 (0%)	1 (3.23%)	1
*Streptococcus vestibularis*	0 (0%)	1 (3.23%)	1	0 (0%)	1 (3.23%)	1
*Trueperella bernardiae*	0 (0%)	2 (6.45%)	1			
*Veillonella parvula*				0 (0%)	2 (6.45%)	0.54
*Weeksella virosa*	1 (6.67%)	0 (0%)	0.33			

Blank indicates not detected

Bonferroni adjusted *p* value 0.000794

Italics indicate significant at the 0.05 threshold

**Fig. 2:**
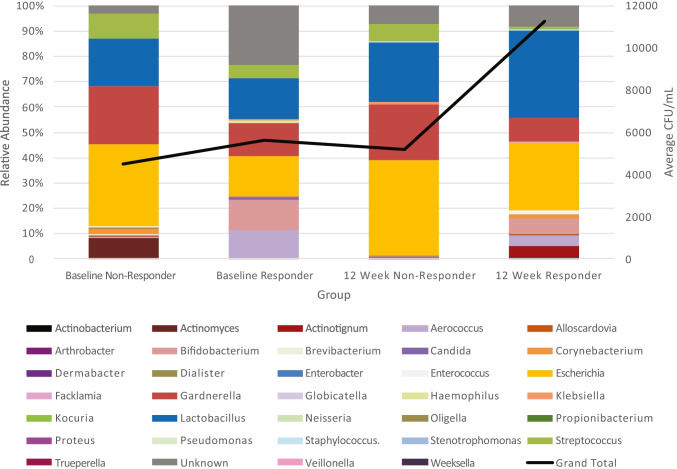
Composite microbiota profiles of responders and nonresponders. Relative abundance of the genera detected in responders and nonresponders at baseline and at 12 weeks

Some of these differences can be seen in the composite microbiota profiles of responders and nonresponders (Fig. 2). For example, it appears that responders were more likely to have the genus *Aerococcus* at baseline and at the 12-week follow-up. The relative abundance of *Aerococcus* appeared to decrease from baseline to the 12-week follow-up, as did the “Other” category, which contains all taxa present at less than 1% relative abundance. In contrast, the relative abundances of the genus *Lactobacillus* increased from baseline to the 12-week follow-up.

## Discussion

Our findings document longitudinal urobiome changes associated with symptom improvement in adult women being treated with mirabegron for UUI. Consistent with clinical experience, patients with milder symptoms were more likely to report satisfactory treatment response. However, in these responders, the mechanism for symptom improvement warrants further study to evaluate whether detected urobiome changes are due to direct effects of mirabegron or related to the changes in urgency and or urinary frequency. Given the well-recognized natural history of UUI, with waxing and waning of symptoms, longitudinal studies of untreated adult women will also be needed as comparator groups to determine the effect of symptom severity on urobiome characteristics. We recommend that larger studies be prioritized to determine whether there are direct effects of these medications on urobiome community characteristics.

This study did not detect baseline urobiome differences by response group. This contrasts with findings from a prior study suggesting that baseline characteristics of the urobiome were associated with dose-related treatment responses to solifenacin, an antimuscarinic commonly used to treat UUI [1]. The lack of baseline differences between response groups suggests that urobiome correlates for mirabegron differ from those associated with solifenacin. The role of bladder control medication on the urobiome is poorly understood. These therapies may vary by medication class in their ability to alter urobiome community characteristics.

Emerging evidence is becoming available about the urobiome in adult women without lower urinary symptoms [7]. Population studies that relate characteristics of the urobiome community to various bladder health states are not yet available; however, the increased diversity of the responder urobiome suggests that this urobiome characteristic might be more closely associated with a preferred bladder health state. Although our small sample size limits conclusive statements regarding individual microbes, the detected presence of the genus *Aerococcus* and several species (*Actinomyces neuii*, *Corynebacterium aurimucosum*, *Lactobacillus gasseri* and *Streptococcus anginosus*) deserves further study.

Although this study was not designed to assess medication efficacy, the “real world” efficacy of 19% suggests opportunities to improve treatment of UUI. This also advances the conversation about the likely heterogenicity of UUI. Although the baseline urobiome did not identify a group of likely responders, further studies are needed to reduce the clinical need to provide medication to 5 patients to successfully help symptoms in only one patient. In addition, medication intolerance (17%) limited the opportunity to determine efficacy in this subgroup.

This study has multiple strengths, including rigorous characterization of participants with validated questionnaires and the use of catheterized urine specimens for urobiome characterization. The latter allows us to focus our characterization on the urobiome of the bladder, which is what we wish to measure. However, we believe that any bias introduced by urinary catheterization affected participations similarly based on the similarities of the pre-treatment urobiome of responders and nonresponders.

Limitations of this study include the small sample size, especially with small numbers of racial and ethnic minority individuals. Second, recruitment from a single center limits generalization. Third, consistent with observations from clinical practice, a high proportion of individuals who initiated UUI treatment with medication chose not to continue this therapy. Fourth, we are unable to comment on urobiome characteristics for those participants whose urine sample was EQUC negative. Fifth, treatment response was associated with baseline symptom severity, limiting insights into treatment response for more severely affected patients.

In conclusion, symptom improvement with mirabegron for UUI treatment in adult women is associated with changes in the urobiome after 12 weeks of treatment. With increasing clinical use of beta-3 agonists for UUI symptom relief, further investigation of urobiome characteristics may advance our understanding of therapeutic benefit and ancillary strategies to optimize the urobiome to enhance medication efficacy.
